# Celecoxib in the treatment of orofacial pain and discomfort in rats subjected to a dental occlusal interference model

**DOI:** 10.1590/acb370506

**Published:** 2022-08-15

**Authors:** Andrea Whitehurst Ary Leitão, Marcela Maria Fontes Borges, Joyce Ohana de Lima Martins, Antônio Alexandre Coelho, Anna Clara Aragão Matos Carlos, Ana Paula Negreiros Nunes Alves, Paulo Goberlânio de Barros Silva, Fabrício Bitu Sousa

**Affiliations:** 1 Master. Centro Univeristário Christus – Department of Dentistry – Laboratory of Oral Pathology – Fortaleza (CE), Brazil.; 2 Fellow Master degree. Universidade Federal do Ceará – Faculty of Pharmacy, Dentistry and Nursing – Division of Oral Pathology – Fortaleza (CE), Brazil.; 3 Graduate student. Centro Univeristário Christus – Department of Dentistry – Fortaleza (CE), Brazil.; 4 Dentist. Centro Univeristário Christus – Department of Dentistry – Fortaleza (CE), Brazil.; 5 PhD, Full Professor. Universidade Federal do Ceará – Faculty of Pharmacy, Dentistry and Nursing – Division of Oral Pathology – Fortaleza (CE), Brazil.; 6 PhD, Full Professor. Centro Univeristário Christus – Department of Dentistry – Laboratory of Oral Pathology – Fortaleza (CE), Brazil.

**Keywords:** Cyclooxygenase 2, Dental Occlusion, Facial Pain, Trigeminal Ganglion, Rats

## Abstract

**Purpose:**

To evaluate the effect of a selective cyclooxygenase 2 (COX-2) inhibitor on trigeminal ganglion changes and orofacial discomfort/nociception in rats submitted to an experimental model of dental occlusal interference (DOI).

**Methods:**

Female Wistar rats (180-200 g) were divided into five groups: a sham group (without DOI) (n=15); and four experimental groups with DOI treated daily with 0.1 mL/kg saline (DOI+SAL), 8, 16, or 32 mg/kg celecoxib (DOI+cel -8, -16, -32) (n=30/group). The animals were euthanized after one, three, and seven days. The bilateral trigeminal ganglia were analyzed histomorphometrically (neuron cell body area) and immunohistochemically (COX-2, nuclear factor-kappa B [NFkB], and peroxisome proliferator-activated receptor-y [PPARy]). A bilateral nociception assay of the masseter muscle was performed. The number of bites/scratches, weight, and grimace scale scores were determined daily. One-way/two-way analysis of variance (ANOVA)/Bonferroni post hoc tests were used (P < .05, GraphPad Prism 5.0).

**Results:**

DOI+SAL showed a reduction in neuron cell body area bilaterally, whereas DOI+cel-32 exhibited a significative increase in neuron cell body area compared with DOI+SAL group (P < 0.05). The ipsilateral (P=0.007 and P=0.039) and contralateral (P < 0.001 and P=0.005) overexpression of COX-2 and NFkB and downregulation of PPARy (P=0.016 and P < 0.001) occurred in DOI+SAL, but DOI+cel-32 reverted this alteration. DOI+SAL showed increase in isplateral (P < 0.001) and contralateral (P < 0.001) nociception, an increased number of bites (P=0.010), scratches (P < 0.001), and grimace scores (P=0.032). In the group of DOI+cel-32, these parameters were reduced.

**Conclusions:**

Celecoxib attenuated DOI-induced transitory nociception/orofacial discomfort resulting from trigeminal COX-2 overexpression.

## Introduction

The orofacial region is one of the most innervated areas of the body, rendering the management of pain associated with the trigeminal system, such as migraine, headache, temporomandibular joint disorder, and trigeminal neuralgia quite challenging. Consequently, orofacial pain is one of the most prevalent and debilitating pain conditions, generating significant mood disturbances and neurosensory changes^1^.

Various inflammatory chemical mediators such as prostaglandins are present in the inflammatory process in peripheral nerves, which contribute to the spread of orofacial pain^2^. Cyclooxygenase 2 (COX-2) is an enzyme that can affect nociceptive thresholds and inflammatory pain-related symptoms^3^. Changes in the occlusal pattern may lead to an inflammatory process in periodontal tissue, whereas inflammatory changes in the trigeminal terminals may affect cell bodies in the trigeminal ganglion^4^-^,^ 5.

Recent studies showed out that occlusal interferences can lead to the development of orofacial pain^6^-^-^ 8. Moreover, changes in occlusal stability can cause alterations in the distribution of occlusal loads, which interfere with the functional dynamics of the temporomandibular joints (TMJ)^9^-^,^ 10.

Given those changes in the occlusal pattern, dental inflammatory processes may trigger in the trigeminal system, altering animal behavior with increased signs of orofacial pain and discomfort^5^, and the control of COX-2 expression can attenuate this process^11^. The purpose of the present study was to evaluate the effect of celecoxib (a selective COX-2 inhibitor) on trigeminal ganglion changes and orofacial discomfort/nociception in rats submitted to an experimental model of dental occlusal interference (DOI).

## Methods

### Animals, sample size, and experimental groups

This study was approved by the Ethics Committee on Animal Experimentation (Protocol 036/18, Centro Universitário Christus, Fortaleza, CE, Brazil) and was consistent with the Ethical Guidelines of the International Association for the Study of Pain.

Experiments were performed on a total of 160 adult female Wistar rats (*Rattus norvegicus*) weighing 180-200 g. The rats were housed five per cage in polypropylene cages, fed with water and food *ad libitum* and maintained in a 12-hour light/dark cycle at 20-25°C, being weighed daily. All efforts were made to provide the animals adequate treatment, such as comfortable housing with conspecifics, appropriate care, and handling in the research facilities according to the recommendations of the National Council of Control of Experimental Animals (CONCEA) and to minimize the number of animals used.

Based on the study by Ahn *et al*.^12^, in which rats were treated with an experimental COX-2 selective inhibitor^13^, resulting in a significant reduction in the number of scratches and duration of scratching after formalin injection in the TMJ (123 ± 53 seconds vs. 55 ± 43), a total of 10 animals/group was estimated as necessary to obtain a sample with 90% power and 95% confidence to reject the null hypothesis.

The rats were randomly (Random Command, Microsoft Excel^®^) divided into five groups:

- A sham group submitted to the simulation of the experimental model and treated daily with 0.1 mL/kg saline solution (n=15);
- A negative control group submitted to the DOI model treated daily with 0.1 mL/kg saline solution (DOI+SAL) (n=30);
- Three experimental groups submitted to the DOI model with treated daily with celecoxib 8 (DOI+cel-8), 16 (DOI+cel-16) or 32 (DOI+cel-32) mg/kg (n=30/group).

The doses were based on the study by Gonçalves *et al*.^14^ (16 mg/kg) to construct a dose-response curve with two times higher and lower doses.

Two experimenters were employed: one was responsible for the experimental procedures, and the other one for the histological analysis and the behavior assessment. Therefore, the experimenter responsible for evaluating the animals was always blind to the animal treatment.

### Experimental protocol

The gavage administration of saline solution or celecoxib was performed 1 hour before the procedure. Celecoxib (Eurofarma^®^, Sao Paulo, Brazil) capsules were dissolved in sterile saline solution in a volume of 0.1 mL/kg, and the same volume was administered to all animals. Sham and control groups were administered the same equivalent volume of saline. The drug administration continued daily, once a day, until the end of each protocol, and was performed with the rat inside the cage.

The occlusal interference protocol was adopted. Briefly, an occlusal interference device (OID) was previously manufactured with composite resin (Z350 3M^®^) in a single increment with measurements of 100 × 20 × 1.3 mm (length × width × thickness), which was manually performed in a standardized fashion. After light-curing for 40 seconds (Poly, Wireless, Kavo^®^), the edges of the devices were adjusted to the same thickness. Device thickness was measured with a 0.05 mm precision digital caliper (Lorben^®^) and did not differ significantly among the groups (DOI+SAL0.89 ± 0.013 mm; DOI+Cel-8:0.88 ± 0.013 mm; DOI+Cel-16:0.88 ± 0.012 mm; DOI+Cel-32:0.87 ± 0.014; p=0.890).

After anesthesia (xylazine-20 mg/kg; ketamine-80 mg/kg), phosphoric acid 37% (DFL^®^) was applied on the occlusal surface of the upper left molars (40 seconds) and removed with a piece of gauze soaked in water. The surface was then dried with a new piece of gauze, and the adhesive system (3M^®^, universal) was applied on the entire surface of the etched teeth with individual and sterile microbrushes, followed by light-curing (20 seconds). A thin layer of flow resin (Oppalis Flow, FGM^®^) was applied to the adhesive surface, and the previously manufactured occlusal device was placed on top of the resin. After light-curing (40 seconds), the stability of the device was checked, and the animals were housed in polypropylene cages and monitored until regaining consciousness.

The animals were euthanized by anesthetic overdose (xylazine-60 mg/kg; ketamine-240 mg/kg) after one, three, and seven days of the premature contact to perform the surgical excision of the trigeminal nerve ganglia for histological processing.

### Histological processing and histomorphometric analysis of the trigeminal nerve ganglion

After fixation, left (ipsilateral) and right (contralateral) trigeminal nerve ganglia were histologically processed. Samples were cut into 3-μm thick sections and stained with hematoxylin and eosin (HE). Five microfields (400×) of each slide were photographed with a digital camera (U-TV0.63XC, Olympus^®^) coupled to an optical microscope (BX43, Olympus^®^) with the Olympus Soft Imaging LCMicro software (Olympus^®^) and exported to ImageJ^®^ for histomorphometric analysis. A trained researcher manually measured the area of each cell body, and the mean area of the cell body was adopted as the sampling unit.

### Immunohistochemical technique and analysis

Samples of trigeminal ganglia were cut (3 μm) and deposited on silanized slides. After deparaffinization and rehydration, antigen retrieval was performed with citrate buffer pH 6. After cooling, to inactivate endogenous peroxidase, samples were incubated (30 min) with 3% H_2_O_2_ in phosphate-buffered saline (PBS), washed with PBS, and incubated overnight with primary antibodies directed against anti-Cox2 (1:300, monoclonal, Abcam^®^, ab15191), anti-PPARγ (1:1.500, polyclonal; Thermo Fisher^®^ pa,1824), and anti-NFkB p65 (1:200, monoclonal; Abcam^®^ ab,16502).

After washes in PBS, samples were incubated with Envision Plus HRP anti-rabbit/mouse IgG for 30 min (Dako^®^ K4065), washed again in PBS, and diaminobenzidine chromogen (Dako^®^ K3469) was applied to the samples for 5 min. Harris hematoxylin was used as the counterstain (10 s), and after dehydration and diaphanization, the slides were mounted with Enthelam^®^. Parallel negative control sections were treated with antibody diluent instead of a primary antibody.

Five microfields (400×) of each slide in regions with higher concentrations of neuron cell bodies were photographed with a digital camera (U-TV0.63XC, Olympus^®^) coupled to an optical microscope (BX43, Olympus^®^) and exported to ImageJ^®^. A trained researcher manually counted the number of neuron cell bodies with positive (brownish pigmentation) and negative immunostaining for each marker. The percentage of immunopositive cells was the sampling unit^5^.

### Nociception assay and behavioral study

The animals from the sham (n=5), DOI+SAL (n=10), and DOI+cel-32 (n=10) group underwent nociception assay and behavioral study. Two days before the device installation, the animals were assessed for biting and scratching patterns, Grimace scales, and nociception assay by digital algometry. After the occlusal device installation, the animals were equally and individually conditioned in a dark room with a red light in a polypropylene cage.

After 5 minutes of acclimatization, the rats were observed for another 5 minutes (timed), and the number of bites and scratches, as well as the position and shape of the whiskers, were evaluated^5^. During this time, an assistant used a rat grimace scale to observe behaviors such as orbital tightening, nose/cheek flattening, ear changes, and whisker changes to classify the level of pain and suffering as:

- 0: no pain/suffering;
- 1: mild pain/suffering;
- 2: pain/suffering (severe suffering).

The sum of the scores for each animal (0-8) was used as the sampling unit^5^.

After the pain and suffering assessment, a nociception test was performed using a digital analgesimeter (Bronther^®^) with a transduction capacity of 0.1 to 1,000 g (approximately 1 mN to 10 N), resolution of 2 mV/V, reaction time between 1-150 msec, and temperature range of 10-60°C. A previously trained operator held the animal in ventral decubitus and, once it was immobilized in this position, a von Frey filament was used to stimulate the masseteric region of the animal. This test measures the force (in newtons) the animal can withstand until it develops an escape mechanism. The analysis was repeated three times on each side (ipsilateral and contralateral), and the mean value of the three measurements was used as the sampling unit. The experiment was repeated until the day of euthanasia (seven days after the OID installation)^15^. Additionally, the animals were weighed to assess body mass variation throughout the study.

### Statistical analysis

The data were submitted to the Shapiro-Wilk’s normality test, expressed as mean ± standard error of the mean values, and compared using one-way or two-way analysis of variance (ANOVA) for repeated measures, followed by Bonferroni post hoc test (parametric data). All analyses were performed using the GraphPad Prism 5.0^®^ statistical software, considering a 95% confidence interval level (p < 0.05).

## Results

### Histomorphometric analysis

**Figure 1:**
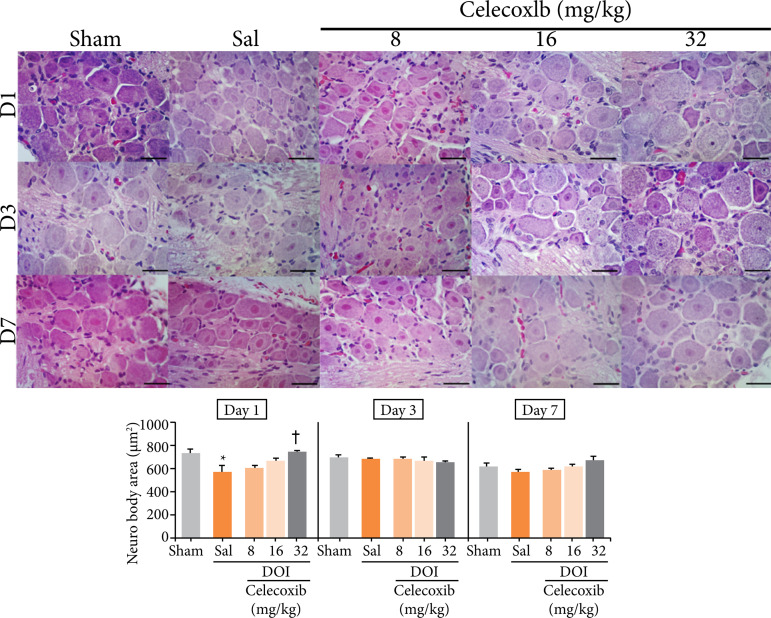
Histomorphometric analysis of ipsilateral cell body area of trigeminal neurons in rats treated with different doses of a selective cyclooxygenase 2 inhibitor (celecoxib, Eurofarma^®^) and exposed to an experimentalmodel of dental occlusal interference. Two-way analysis of variance (ANOVA)/Bonferroni(mean ± standard error); hematoxylin and eosin, 400×, horizontal line = 50 μm.

In the trigeminal ganglia of the ipsilateral side, one day after the installation of the OID, the group DOI+SAL showed a significant reduction in the mean area of the neuron bodies compared to the sham group. The DOI+cel-32 showed a significant increase in the mean area of the neuron bodies compared to the DOI+SAL group (p < 0.001). On days 3 (p=0.878) and 7 (p=0.166) after the installation of OID, there were no significant differences (Fig. 1).

*P < .05 *vs*. sham group on the same day; †P < .05 vs saline group on the same day; DOI: dental occlusal interference.

**Figure 2:**
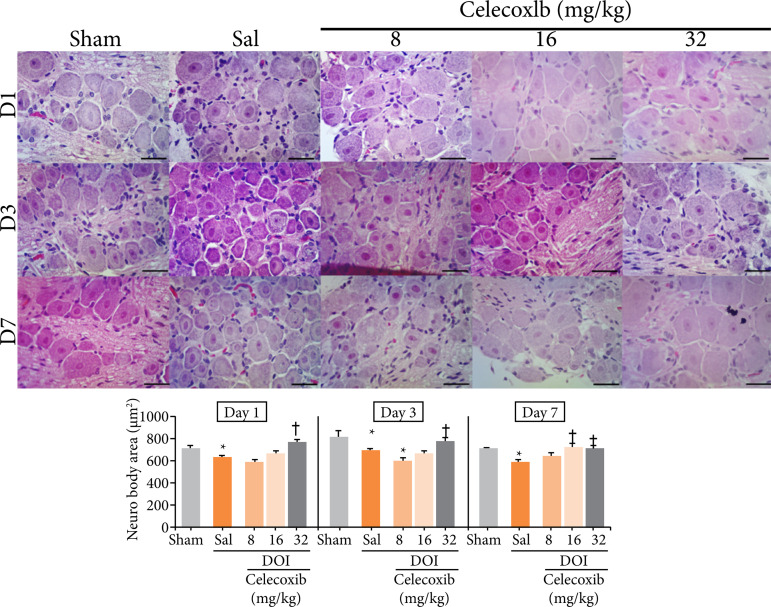
Histomorphometric analysis of contralateral cell body area of trigeminal neurons in rats treated with different doses of a selective cyclooxygenase 2 inhibitor (celecoxib, Eurofarma^®^) and exposed to an experimentalmodel of dental occlusal interference. Two-way analysis of variance (ANOVA)/Bonferroni(mean ± standard error); hematoxylin and eosin, 400×, horizontal line = 50 μm.

In the contralateral ganglia one and three days after the installation of the OID, the DOI+SAL group and the DOI+cel-8 group showed a significant reduction in neuron body area compared to the sham group, whereas the DOI+cel-32 group showed a significant increase of the neuron cell body area compared to DOI+SAL group (p=0.001 and p < 0.001, respectively). After seven days, only the DOI+SAL group showed a significant reduction in neuron cell body area compared to the sham group, and the neuron cell bodies of the groups treated with celecoxib 16 and 32 mg/kg were comparable to those of the sham group, and change significantly with the DOI+SAL (p=0.001) (Fig. 2).

*P < 0.05 *vs*. sham group on the same day; †P < .05 vs saline group on the same day; DOI: dental occlusal interference.

**Figure 3:**
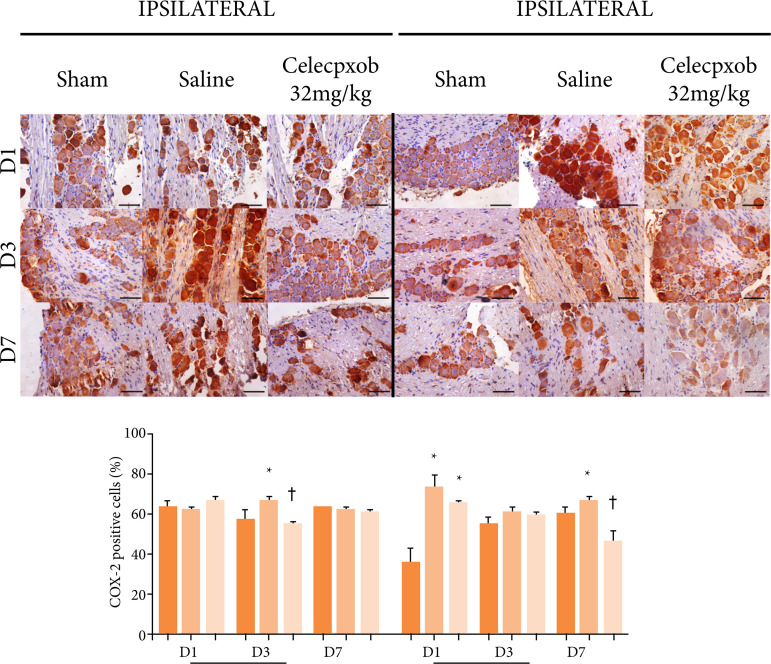
COX-2 immunostaining of cell bodies of trigeminal neurons after one, three, and sevendays of exposure to an experimental model of dental occlusal interference in rats treated witha selective COX-2 inhibitor (celecoxib, Eurofarma^®^). One-way ANOVA/Bonferroni(mean ± standard error). Immunohistochemistry, 400×, horizontal line = 50 μm.

There was no difference in COX-2 expression one (p=0.567) and seven (p=0.497) days after OID installation in the ipsilateral ganglia. However, after day 3, in comparison with the sham group, the saline-treated animals demonstrated a significant increase in the immunostaining for COX-2, whereas the DOI+cel-32 group exhibited a significant reduction in the immunostaining for COX-2 when compared to DOI+SAL group (p=0.007) (Fig. 3).

COX-2: cyclooxygenase 2; white bar: sham group; gray bar: DOI+SAL group; black bar: DOI+cel-32 group;*P < .05 *vs*. sham group; †P < .05 *vs*. saline; ANOVA: analysis of variance.

### Immunohistochemical analysis

In the trigeminal ganglia on the contralateral side, on day 1 after the DOI model procedure, the DOI+SAL and DOI+cel-32 groups manifested a significant increase in COX-2 immunostaining compared to the sham group (p < 0.001). Three days after the installation of the OID, there was no significant difference among the groups (p=0.327). After seven days, the saline group showed a significant increase in COX-2 immunostaining, and the DOI+cel-32 demonstrated a significant reduction in comparison with the DOI+SAL group (p=0.013) (Fig. 3).

**Figure 4:**
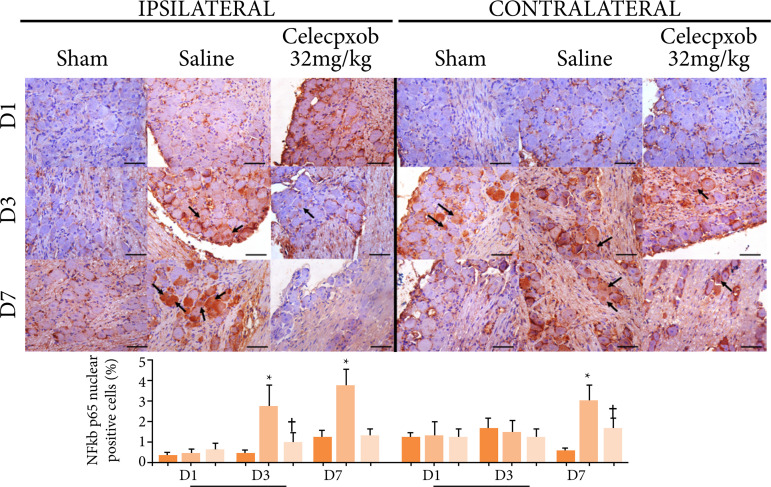
NFkB p65 immunostaining of cell bodies of trigeminal neurons after one, three, and sevendays of exposure to an experimental model of dental occlusal interference in rats treated witha selective cyclooxygenase 2 inhibitor (celecoxib, Eurofarma^®^). One-way ANOVA/Bonferroni(mean ± standard error). Immunohistochemistry, 400×, horizontal line = 50 μm.

In ipsilateral ganglia, there was no statistical difference in nuclear NFkB immunostaining after one day (p=0.635) of the OID installation. After three (p=0.039) and seven (p=0.005) days, compared to the sham group, the animals that received saline solution exhibited a significant increase in the percentage of neuron cell bodies expressing nuclear NFkB, whereas the group treated with celecoxib 32 mg/kg exhibited a significant decrease compared with DOI+SAL. On the contralateral side, there was no statistical difference in nuclear NFkB expression after one (p=0.994) and three (p=0.863) days. However, on day 7, the DOI+SAL group showed a significant increase in nuclear immunostaining of NFkB, and the DOI+cel-32 group showed a significant decrease (p=0.005) compared to the DOI+SAL (Fig. 4).

NFkB: nuclear factor-kappa B; white bar: sham group; gray bar: DOI+SAL group; black bar: DOI+cel-32 group;*P < .05 *vs*. sham group; †P < .05 *vs*. saline; ANOVA: analysis of variance.

**Figure 5:**
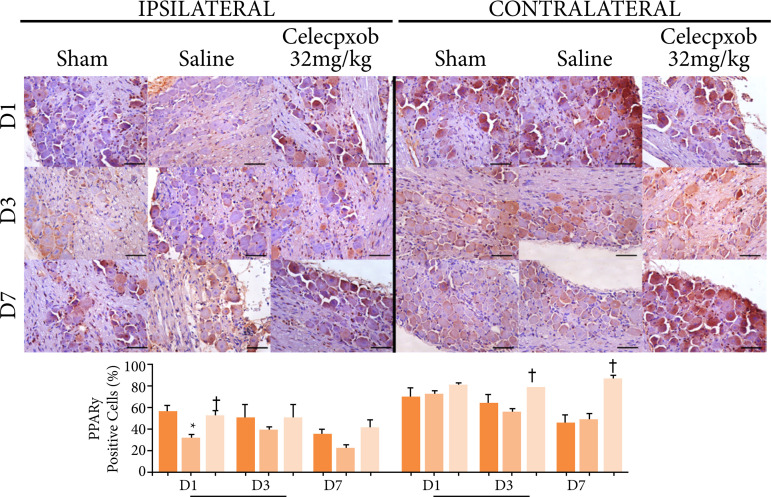
Peroxisome proliferator-activated receptor-y immunostaining of cell bodies of trigeminal neurons afterone, three, and seven days of exposure to an experimental model of dental occlusal interference in ratstreated with a selective cyclo-oxygenase-2 inhibitor (celecoxib, Eurofarma^®^). One-way analysis ofvariance/Bonferroni (mean ± standard error). Immunohistochemistry, 400×, horizontal line = 50 μm.

On the ipsilateral ganglia, one day after the experimental model procedure, the saline group showed a significant reduction in the percentage of immunostaining for PPARy compared to the sham group, and the DOI+cel-32 group showed a significant increase compared with DOI+SAL group (p=0.016). After three (p=0.668) and seven (p=0.168) days, there was no difference among the groups. On the contralateral ganglia, there was no difference in PPARy immunostaining on the first day after the OID installation (p=0.688). After three days, the saline group showed a significant reduction in the PPARy immunostaining compared to the sham group, while DOI+cel-32 group showed a significant increase compared with the DOI+SAL group (p=0.037). Seven days after the implementation of the DOI protocol, there was no significant difference in the immunostaining for PPARy in the sham and saline groups. The group treated with celecoxib 32 mg/kg showed a significant increase in the expression of this immunomarker compared to the saline and sham groups (p < 0.001) (Fig. 5).

*P < .05 *vs*. sham group on same the day; †P < .05 *vs*. saline group on the same day; ANOVA: analysis of variance.

### Nociception assay and behavioral study

There was a significant increase in the mean number of bites in the animals submitted to the DOI model. Five and six days after performing the DOI procedure, the animals treated with saline solution showed a significant increase in the number of bites compared to the sham group and with the group that received treatment with celecoxib 32 mg/kg (p=0.010). The results of the mean number of scratches were similar on days 4, 5, and 6 after the placement of the OID. The saline-treated group showed an increase in these values compared to the sham group and the groups treated with celecoxib (p < 0.001) (Table 1).

The grimace scores of the sham group were 0, as the animals exhibited no signs of distress on any of the days during the experimental protocol. The group gavaged with saline solution and submitted to the DOI model showed a mean increase in grimace scores after three, four, and six days of the OID installation. Treatment with celecoxib 32 mg/kg exhibited no statistical difference compared to the sham group (p=0.032) (Table 1).

The nociceptive threshold showed a significant reduction in the group submitted to the DOI model compared to the sham group on both the ipsilateral and contralateral sides. On the ipsilateral side, the group treated with saline showed a significant reduction in the nociceptive threshold from day 4 to the end of the experimental protocol. In the group treated with celecoxib, a decrease in the nociceptive threshold was observed only on the sixth and seventh days after the installation of the OID compared to the sham group (p < 0.001). On the contralateral side, the group treated with saline showed a significant reduction in the nociceptive threshold from the third day until the end of the experimental protocol. In the group treated with celecoxib, a decrease in the nociceptive threshold also happened only on the sixth and seventh day compared to the sham group (p < 0.001). There was no significant difference in the body mass of the animals in the three experimental groups (p=0.613) (Table 1).

**Table 1:** Temporal course of nociception assay, counting of bites/scratches, mensuration of Grimace scale and weight loss in rats treated with celecoxib (Eurofarma^®^) and exposed to experimental model of occlusal dental interference.

Parâmetros comportamentais		Time (Days)							p-Value
		1	2	3	4	5	6	7	
Bites/ 5 min									
	Sham	12.63 ± 5.78	9.00 ± 4.03	12.75 ± 5.26	7.63 ± 2.37	4.75 ± 1.85	3.63 ± 1.52	6.83 ± 3.70	0.010
	Saline	24.00 ± 10.62	38.13 ± 25.27	31.00 ± 19.49	24.88 ± 16.17	51.38 ± 15.30*	55.63 ± 24.17*	26.38 ± 7.02	
	Celecoxib	16.63 ± 9.73	40.25 ± 26.79	25.25 ± 7.87	27.25 ± 6.13	12.00 ± 3.51	15.38 ± 5.11	13.75 ± 6.00	
Scratches/ 5 min									
	Sham	4.38 ± 2.06	3.83 ± 2.32	2.50 ± 0.89	1.86 ± 0.67	0.43 ± 0.43	0.88 ± 0.44	1.86 ± 0.96	< 0.001
	Saline	5.00 ± 2.44	1.38 ± 0.91	4.88 ± 3.11	19.00 ± 5.95*	12.13 ± 5.99*	11.75 ± 7.80*	5.50 ± 2.78	
	Celecoxib	2.00 ± 0.91	2.67 ± 1.26	2.29 ± 1.06	2.86 ± 1.32	2.57 ± 1.13	1.71 ± 0.87	4.63 ± 1.60	
Grimmace scale sum (0-8)									
	Sham	0.00 ± 0.00	0.00 ± 0.00	0.00 ± 0.00	0.00 ± 0.00	0.00 ± 0.00	0.00 ± 0.00	0.00 ± 0.00	0.032
	Saline	0.00 ± 0.00	0.00 ± 0.00	0.38 ± 0.26*	0.25 ± 0.16*	0.13 ± 0.13	0.25 ± 0.25*	0.00 ± 0.00	
	Celecoxib	0.00 ± 0.00	0.13 ± 0.13	0.13 ± 0.13	0.00 ± 0.00	0.00 ± 0.00	0.00 ± 0.00	0.00 ± 0.00	
Nociception ipsilateral masseter (N)									
	Sham	44.38 ± 6.44	39.63 ± 5.04	30.75 ± 2.61	43.25 ± 4.20	40.75 ± 4.68	62.50 ± 6.86	50.38 ± 8.37	< 0.001
	Saline	48.13 ± 7.56	26.38 ± 2.58	17.50 ± 2.53	19.00 ± 3.84*	23.25 ± 2.15*	23.00 ± 2.04*	23.25 ± 3.89*	
	Celecoxib	53.13 ± 7.97	34.13 ± 4.79	22.00 ± 2.55	28.13 ± 4.51	28.50 ± 2.33	30.88 ± 4.55*	27.75 ± 4.05*	
Nociception contra lateral masseter (N)									
	Sham	38.63 ± 4.54	39.50 ± 5.96	33.75 ± 3.06	37.00 ± 4.79	38.75 ± 4.92	44.38 ± 7.81	41.50 ± 5.44	< 0.001
	Saline	32.50 ± 2.33	25.25 ± 2.27	15.25 ± 1.22*	21.00 ± 2.63*	21.50 ± 3.77*	27.75 ± 3.38*	23.38 ± 2.44*	
	Celecoxib	36.38 ± 4.40	23.75 ± 2.35	22.38 ± 2.51	30.63 ± 4.83	24.63 ± 5.06	29.25 ± 2.66*	27.75 ± 3.38*	
Weight (%)									
	Sham	100.00 ± 0.00	98.65 ± 0.85	99.40 ± 0.71	99.00 ± 0.66	98.88 ± 0.77	99.06 ± 1.51	101.93 ± 1.29	0.613
	Saline	100.00 ± 0.00	96.27 ± 1.24	97.34 ± 1.12	96.98 ± 1.57	97.01 ± 1.58	97.00 ± 1.28	100.50 ± 2.02	
	Celecoxib	100.00 ± 0.00	96.97 ± 0.99	97.60 ± 0.79	97.13 ± 0.47	97.58 ± 0.74	98.34 ± 0.60	101.36 ± 0.61	

* p < 0.05 versus sham in same day.

## Discussion

In the present study, behavioral and nociceptive changes were observed in animals submitted to an experimental model of DOI, demonstrating an association with COX-2-dependent neuroinflammatory changes in the trigeminal ganglion. Treatment with celecoxib attenuated behavioral, nociceptive, and histomorphometric changes, while also increasing the expression of neuroprotective transcription factors (PPARy).

COX-2-related prostaglandins, such as PGE2, stimulate neuronal sensitization of sensory neurons^16^-^-^ 19. This neuroinflammatory process was observed in the nerve terminals of the trigeminal nerve located in the apical region of teeth submitted to DOI^10^, which increased the immunoexpression of COX-2 in the trigeminal ganglion, resulting in inflammatory stress in this set of neurons^17^-^,^ 18.

COX-2 can be induced by several transcription factors, and a direct relationship with NFkB has been previously described^19^. COX-2 is one of the targets of NFkB activation, and nuclear activation of this transcription factor induces COX-2-dependent neuroinflammation^20^. On the other hand, the resolution of the neuronal inflammatory process has been associated with a reduced immunoexpression of NFkB and increased immunoexpression of PPARy.

Peroxisome proliferator-activated receptors (PPARs) belong to a family of nuclear gene transcription factors whose gamma isoform (PPARy) is the most prevalent in the nervous system. When activated, these receptors exhibit antioxidant and anti-inflammatory activity by blocking NFkB activation and suppressing neuroinflammation^21^. PPARy reduces transcriptional activity and blocks the production of numerous cytokines such as TNF-α^22^-^,^ 23, which is strongly related to neuronal and behavioral changes in the head and neck region^5^ and has been linked to neuroprotective actions, as reported by studies of neurodegenerative diseases (Parkinson’s, Alzheimer’s and Huntington’s)^24^.

Central sensitization mechanisms are involved in maintaining the mechanical hyperalgesia induced by DOI, which is directly related to masticatory muscle pain^25^-^,^ 26. DOI induces inflammation in the peripheral nervous tissues, resulting in sensitization of trigeminal sensory neurons and hyperalgesia. In other words, the overlap of underlying mechanisms contributes to the spread of orofacial pain^27^, as similarly demonstrated in the DOI model adopted in this study.

The mechanical stress generated by malocclusions stimulates prostaglandin production by various local cells^28^, and we observed that blocking this pathway was critical in reducing orofacial nociception. Zhang *et al*.^29^ described that inflammatory TMJ pain was regulated by COX-2, which makes non-steroidal anti-inflammatory drugs one of the main pharmacological approaches for the treatment of temporomandibular dysfunctions and head and neck pain^30^.

The results of animal studies cannot always be extrapolated to the clinical setting, and the dimensions of the OID proportionally magnify the pathological changes that occur in humans as a result of occlusal interference.

## Conclusions

In the present study, the installation of a device to simulate premature occlusal contact led to significant trigeminal ganglion neuroinflammatory changes, and treatment with celecoxib partially reversed these changes. The findings in this study reinforce the role of the COX-2 pathway in head and neck neuroinflammation and may guide investigations in other regions innervated by branches of the trigeminal ganglion.
